# The effect of jujube powder incorporation on the chemical, rheological, and sensory properties of toffee

**DOI:** 10.1002/fsn3.912

**Published:** 2018-12-19

**Authors:** Mahdieh Bahrasemani Koohestani, Mohammad A. Sahari, Mohsen Barzegar

**Affiliations:** ^1^ Department of Food Science and Technology Faculty of Agriculture Tarbiat Modares University Tehran Iran

**Keywords:** jujube powder, physico‐chemical, rheological, sensory properties, toffee

## Abstract

One of the major problems caused by the consumption of toffee is its high sugar content. So, great efforts have been made to replace sugar in toffee with bioactive ingredients. To the best of our knowledge, there is no research relevant to the application of jujube powder as sugar replacer in toffee. In this study, the sugar content of the toffee was replaced with the jujube powder in six different levels 0%, 20%, 40%, 60%, 80%, and 100%, and the physico‐chemical, rheological, and sensory properties of the toffee were evaluated. The results showed that the fat, raw energy, pH, color (L*, a*, b*), sucrose, and firmness of the toffee samples decreased by increasing the percentage of jujube powder in the formulas but their moisture, water activity, ash, protein, glucose, and fructose increased. The rheological results revealed that storage modulus (*G′*) was greater than loss modulus (*G″*), which represents the elastic behavior (solid‐like) of the samples. Also storage modulus (*G′*) of the control sample (jujube powder instead of 0% sugar) was greater than that the other samples. On the other hand, the firmness of the samples was decreased by increasing the percent of jujube powder. According to the chemical and rheological results, samples III and IV (jujube powder instead of 40% and 60% sugar, respectively) were selected as optimum samples. Then the sensory properties were compared to that of control. No significant difference observed in chewiness, taste, flavor, sweetness, color, after‐taste, and overall acceptance between the control and the two other samples.

## 1 INTRODUCTION

Consuming different confectionary products including toffees is very common throughout the world as they contain a lot of energy and the necessary nutrients (Ahmadnia & Sahari, 2008; Habibi Najafi, Vahedi, Yaghanehzad, & Hoseini, 2011). Toffee is a chewy confection with a soft or hard consistency, which is desirable for different groups of people. Given the growing tendency of consumers toward low‐fat and low‐sugar foods and also the impossibility of removing fat and sugar from many foods like sweets, replacing sugar, and fat in such foods is recommended. In addition to the authorized food sweeteners such as sorbitol, maltitol, acesulfame potassium, and sucralose in the formulation of confectionary products, using sugar‐rich fruits such as jujube, dates, and figs (in the forms of a powder or a concentrate) can be achieved as an alternative to sugar. Crystallization of sugar during storage is one of the most important disadvantages of toffee. This can be reduced by replacing the products’ sucrose with the aforementioned fruits as they contain high saccharide in them. Improving the sensory characteristics of the product, diversification of toffee with different flavors and colors, and also improving the nutritional value of the product in terms of minerals and vitamins C, A, B_1,_ and B_2_ are among the advantages of toffee product (Ahmadnia & Sahari, 2008; Habibi Najafi et al., 2011).

Containing plenty of sugar and, consequently, a lot of calorie (or calory), toffees can cause obesity, diabetes, and cardiovascular diseases (Livesey, 2001). One way to cope with this problem is reducing sugar in the formulation of toffee and replacing it with dried fruits with high sugar content, such as jujube, dates, raisins, and figs.

Jujube, with the scientific name of *Ziziphus jujube Miller*, belongs to Rhamnaceae family. Ziziphus comes from a Hebrew word. The jujube tree, which is one of the native vegetation of Iranian plateau, is found in the provinces of Khorasan, Golestan, Mazandaran, Fars, Isfahan, Yazd, Hamedan, and Kerman; however, South Khorasan Provinces rank is first in terms of acreage and production of jujube in Iran (Shams Najafabadi, Sahari, Barzegar, & Hamidi Esfahani, 2017). Being nutritious and very delicious, jujube is full of vitamin C and amino acids (Wang et al., 2016). Recent studies have shown that jujube has anti‐cancer, anti‐inflammatory, hepatoprotective, anti‐oxidative, and anti‐insomnia properties, protects the digestive and nervous systems, and stimulates the immune system (Guo et al., 2015).

Various parts of jujube such as bark, leaves, flowers, and fruits contain numerous medicinal properties like contraceptive, analgesic, and anti‐diabetic. The seed kernel extract is used to reduce insomnia and anxiety (Zhang, Jiang, Ye, Ye, & Ren, 2010). Jujube has been used for more than 2000 years in the traditional Chinese medicine (Qu, Yu, Luo, Zhao, & Huang, 2013), mostly as a treatment for tumors and cardiovascular diseases. Recently, the antioxidant activity of different parts of the fruit, including skin, pulp, and seed, has been reported, which is attributed to the high levels of phenolic compounds. Jujube contains significant amounts of phenolic compounds such as chlorogenic, gallic, and caffeic acid (Shams Najafabadi et al., 2017; Zhang et al., 2010). In this study, the powder of jujube fruit was produced and used instead of 0%, 20%, 40%, 60%, 80%, and 100% sugar in toffee formulation. Then the physico‐chemical, rheological properties were investigated, and finally the sensory properties of the produced toffee in optimum samples were compared.

## 2 MATERIALS AND METHODS

### 2.1 Raw materials

Fat‐free powdered milk (form Pegah Milk Company), glucose syrup with DE = 42 (from Dextrose Iran Company), Lecithin (form Behpak Company), cocoa butter (from Colombian Luker Company), sugar, and salt bought from local supermarkets were used.

#### 2.1.1 Preparing jujube powder

Jujube fruits were taken from the Research Center and Agriculture Farm in Birjand, Southern Khorasan, Iran.

First, the fruits were washed, and their seeds were separated. Then they were divided into smaller parts and dried in a normal oven at a temperature of 45°C for a week and next in a vacuum oven at a temperature of 30°C for 24 hr to remove the moisture. After getting dried, the moisture of the jujubes decreased from 51.17% to 7%–12%. The samples were left to cool; subsequently, they were ground to a fine powder; and then they were passed through a 70 mesh sieve. Next, they were kept in glass containers with lid in the freezer at a temperature of −20°C until being used in toffees. The samples were kept in freezer to prevent moisture absorption.

#### 2.1.2 Calculating the amount of glucose, fructose, and sucrose in the jujube powder and toffee samples using HPLC method

**Figure 1:**
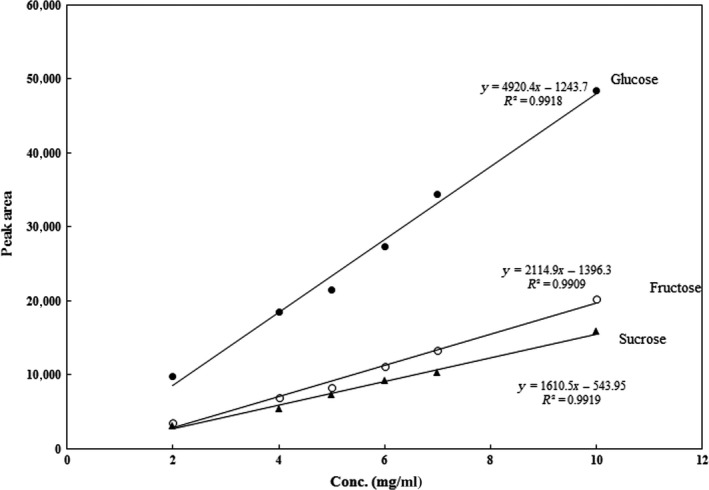
Calibration curve, line equation, and *R* ^2^ in 3 standard sugars (glucose, fructose, and sucrose)

In order to calculate the amount of glucose, fructose and sucrose, 2 g of jujube powder and 2 g of defatted and dried toffee sample were separately combined with 20 ml of distilled water. For defatting purpose, 5 g of dried toffee powder was washed using 50 ml of n‐hexane at three stages and then were sifted using paper filters. The parts remaining on the paper filters were then degreased (Lettieri‐Barbato et al., 2012). After that, the samples were placed at room temperature to dry. Next, they were homogenized at 5,000 rpm for 2 min using an Ultra‐Turrax T‐18 agitator made in Germany (Gao, Wu, Wang, Xu, & Du, 2012). The materials were then centrifuged at 10,000 rpm at ambient temperature for 30 min using Sigma Centrifuge 3‐30 K Model (Germany). The separated supernatants were sifted using a 0.45 μl cellulose filter and then were transferred into vials. They were, then, used as a stock solution. A concentration of 0.1 ppm was taken from the stock solution and injected into the HPLC system. The samples were analyzed using an HPLC system Azura Model made by KNAUER Company in Berlin, Germany. Then 20 μl of the prepared sample was injected for each analysis. The analysis of sugars on the columns was performed at 1 ml/min using Carbopac PA1 (USA). The column's temperature was set to 32°C. The mobile phase was 0.2 M NaOH and distilled water. In order to separate the sugar, the method of isocratic was used in such a way that during the first 15 min, the portion of 0.2 M NaOH and distilled water was 25%:75%, respectively. From 15 to 25 min, NaOH with the ratio of 100% and from 25 to 35 min, NaOH with the ratio of 25% were used as the mobile phase. The overall time of each injection was 35 min. An electrochemical detector Decade Elite Model (Netherlands) was also used. Calibration curve, line equation, and R^2^ in three standard sugars (glucose, fructose, and sucrose) using HPLC method are shown in Figure 1.

#### 2.1.3 Calculation of the amount of replaced sugar with jujube powder in the formulation of toffee

The objective of the study was to determine how much jujube powder should be used so that the consumers are satisfied with the sensory evaluation of the product. The initial evaluation showed that 60% of jujube powder will satisfy the consumers. Thus, 0%, 20%, 40%, 60%, 80%, and 100% of the powder (formula I (jujube powder instead of 0% sugar) = control − VI) was tested and added to the formulation of toffee samples (Ahmadnia & Sahari, 2008).

The basis of calculations in these formulations was replacing in terms of sweetness of the sugars. Since the sweetness values of one gram of sucrose is 100, the values of other sugars are expressed compared to sucrose; therefore, the value of fructose is 180, and the value of glucose is 74. Having had the highest sweetness values, these three sweeteners were used in replacing the jujube powder (Ahmadnia & Sahari, 2008).

Since there is 40.2 g of sugar in the original formulation of toffee, and the sweetness value of sugar is 100, the sweetness value of the product will be 40.2 × 100 = 4,020.

According to the analysis of jujube powder using HPLC, which contains 17.82% of glucose, 21.85% of fructose, and 33.89% of sucrose, the calculation will be as follows:

As 40.2 g of sugar contains the sweetness value of 4,020, such sweetness should be achieved after replacing the jujube powder so that the consumer accepts the product. In other words, the sweetness of the products should be 4,020 in all the formulations. According to the analysis of the compounds of jujube powder, the sweetness value of 100 g of jujube powder is equal to the overall sweetness of glucose, fructose, and sucrose.

The sweetness value of glucose is 17.82 × 74 = 1318.68, the sweetness value of fructose is 21.85 × 180 = 3,933, and that of sucrose is 33.89 × 100 = 3,389.

As a result, the sweetness value of jujube powder is equal to the overall sweetness of fructose, glucose, and sucrose: 1318.68 + 3,933 + 3,389 = 8640.68

Since the objective was to preserve the sweetness value of the product, the replacement of 20% of sugar with jujube powder was done as follows:

**Table:** 

40.2	100
*X* = 8.04	20

This means that 8.04 g of sugar should be replaced with jujube powder that contains the sweetness value of 8.04 × 100 = 804:

Gram of jujube powder = sweetness value

**Table:** 

100	8640.68
*X* = 9.30	804

For replacing 20% sugar, 9.30 g jujube powder is needed (formula II)

The calculations for the content of jujube powder replaced with 40, 60, 80, 100 g of sugar were done the same way as follows:

In formula III, 18.61 g of jujube powder was used, which was equal to 40% of sugar.
In formula IV, 28 g of jujube powder was used, which was equal to 60% of sugar.
In formula V, 37.2 g of jujube powder was used, which was equal to 80% of sugar.
In formula VI, 46.5 g of jujube powder was used, which was equal to 100% of sugar.

#### 2.1.4 Sample preparation

The amounts of the components were optimized in the initial experiments. It was done in such a way that, at the beginning, the basis was the toffee produced by Ahmadnia and Sahari (2008), who used dates as sugar replacer. Then the amount of fat was optimized. Finally, the optimized formula was obtained. The following proportions were obtained (Table 1).

**Table 1:** The content of materials used in the formulation of toffees

Formulation	Ingredient							
	Jujube powder (g)	Sugar (g)	Cocoa butter (g)	Butter (g)	Glucose syrup (g)	Milk powder (g)	Salt (g)	Lecithin (g)
Formula I (Control: jujube powder instead of 0% sugar)	0	40.2	9.3	19.2	4.02	26.68	0.1	0.5
Formula II (jujube powder instead of 20% sugar)	9.30	32.16	9.3	19.2	4.02	26.68	0.1	0.5
Formula III (jujube powder instead of 40% sugar)	18.61	24.12	9.3	19.2	4.02	26.68	0.1	0.5
Formula IV (jujube powder instead of 60% sugar)	28	16.08	9.3	19.2	4.02	26.68	0.1	0.5
Formula V (jujube powder instead of 80% sugar)	37.2	8.04	9.3	19.2	4.02	26.68	0.1	0.5
Formula VI (jujube powder instead of 100% sugar)	46.5	0	9.3	19.2	4.02	26.68	0.1	0.5

A mixture of sugar, liquid glucose, and some water was melted at 150°C. Then the butter and cocoa butter were added and melted. Next, salt, milk powder, and lecithin were added to the obtained solution. The materials were stirred to reach necessary consistency. When the required consistency was reached and the materials were left to cool down, they were wrapped in parchment papers and aluminum foils and kept in plastic containers with lids in the freezer until they were tested.

#### 2.1.5 Analytical methods

The moisture content (normal oven made by Memmert, Germany; Ahn, Kil, Kong, & Kim, 2014), water activity (Novasina SPRINT–TH 500 Model made in Switzerland), fat content, raw energy (Calorimeter Bomb Gallenkamp Auto bomb Model, UK), ash content, and protein content were tested using standard methods (AOAC 2012), and pH (Arunepanlop, Morr, Karleskind, & Laye, 1996) and color (Color Flex Hunterlab, USA; Leon, Mery, Pedreschi, & Leon, 2006) were measured.

#### 2.1.6 Firmness

The *firmness* of the produced toffee samples was measured using the instron texture analyzer (KN 50 Model made Hounsfield, UK; Bourne, 2002).

#### 2.1.7 Rheological properties

The rheological characteristics of the toffee samples were measured using an Anton Paar MCR 301 Model Rheometer (Germany), equipped with Peltier Plate temperature adjuster and water circulator with an accuracy of ±0.01°C. In order to homogenize and control the temperature, first, the samples were stirred at 45°C at the cutting speed of 5 s^−1^ for 500 s. No point was recorded during this time. Then the cutting speed was increased in the range of 2–50 s^−1^_,_ and 21 points were recorded during 180 s (Glicerina, Balestra, Dalla Rosa, & Romani, 2013).

#### 2.1.8 Sensory evaluation

The sensory evaluation of the toffee samples was done using 30 half‐trained taste evaluators (randomly from students of Food Technology Department) applying 5‐point hedonic scale (Golob, Micovic, Bertoncelj, & Jamnik, 2004). Scores were established as 5 for excellent, 4 for good, 3 for fair, 2 for poor, and 1 for terrible.

#### 2.1.9 Statistical analysis

The analysis of the data was carried out using the SPSS V.18 software. Kruskal–Wallis non‐parametric test was used for evaluating the data. All experiments were conducted in triplicates, and the data presented in tables are expressed as the mean ± standard deviation.

## 3 RESULTS AND DISCUSSION

Some of the physico‐chemical characteristics of jujube powder are presented in Table 2.

**Table 2:** Some of the physico‐chemical characteristics of jujube powder

Parameter	Amounts
Moisture (%)	7.08 ± 0.07
Water activity	0.5 ± 0.01
Fat (%)	0.45 ± 0.05
Energy (kcal/g)	3.79 ± 0.15
Ash (%)	2.09 ± 0.05
Protein (N × 6.25)	3.58 ± 0.2
pH	4.8 ± 0.15
Sugar (%; Glu + Fru + Suc)	73.56 ± 0.37
L*	66.35 ± 0.1
a*	8.26 ± 0.09
b*	28.79 ± 0.15

Note

The amounts given in this table show the average data ± standard deviation obtained from 3 repeats.

The findings showed that b* (yellowness) number is greater than a* (redness), implying that the color combinations of jujube powder are mostly carotenoids (yellow–orange) and flavonoids (yellow; Fennema, 2005).

### 3.1 Results of the tests in produced toffee samples

In the second part of the study, the sugar content was replaced by jujube powder in six levels (0%, 20%, 40%, 60%, 80%, and 100%). Some physic‐chemical characteristics of the toffee samples were compared to the control sample (normal toffee sample without jujube powder).

The results of the moisture tests (Table 3) showed that the moisture has significantly (*p* < 0.05) increased from the control sample to sample V. In addition to the increase of jujube powder, the reason behind such increase might be that some materials such as glucose syrup were there in the formulation of toffee, which has hydrophilic characteristics. Another reason could be sugars with low molecular weights (DE = 42), which highly absorb moisture (Zheng, Jin, & Zhang, 2007). Moreover, simple sugars such as glucose and fructose in jujube powder and glucose in the glucose syrup (used in the formulation of toffee) might be another reason of such increase in the moisture content because these sugars absorb moisture.

**Table 3:** Some of the physico‐chemical characteristics of the toffee samples

Parameter	Formula					
	Formula I (control: jujube powder instead of 0% sugar)	Formula II (jujube powder instead of 20% sugar)	Formula III (jujube powder instead of 40% sugar)	Formula IV (jujube powder instead of 60% sugar)	Formula V (jujube powder instead of 80% sugar)	Formula VI (jujube powder instead of 100% sugar)
Moisture (%)	6.22 ± 0.25^f^	8.07 ± 0.21^e^	9.19 ± 0.11^d^	11.92 ± 0.15^c^	12.37 ± 0.26^b^	13.66 ± 0.06^a^
Water activity (a_W_)	0.33 ± 0.02^e^	0.38 ± 0.01^d^	0.45 ± 0.02^c^	0.57 ± 0.01^b^	0.59 ± 0.01^ab^	0.62 ± 0.02^a^
Fat (%)	26.18 ± 0.21^a^	26.07 ± 0.2^a^	23.4 ± 0.29^b^	22.87 ± 0.19^c^	18.87 ± 0.22^d^	18.30 ± 0.19^e^
Energy (kcal/g)	5.81 ± 0.16^a^	5.57 ± 0.13^ab^	5.41 ± 0.15^b^	5.36 ± 0.24^b^	4.99 ± 0.2^c^	4.89 ± 0.17^c^
Ash (%)	2.3 ± 0.1^e^	2.57 ± 0.04^d^	2.71 ± 0.06^b^	3.19 ± 0.06^b^	3.29 ± 0.04^ab^	3.38 ± 0.1^a^
Protein (N × 6.25)	8.53 ± 0.35^b^	9.8 ± 0.18^a^	9.64 ± 0.31^a^	9.98 ± 0.17^a^	9.89 ± 0.38^a^	10.14 ± 0.37^a^
pH	5.87 ± 0.03^a^	5.87 ± 0.03^a^	5.83 ± 0.03^b^	5.8 ± 0.02^bc^	5.77 ± 0.02^c^	5.70 ± 0.02^d^
L*	62.86 ± 0.02^a^	40.24 ± 0.04^b^	36.68 ± 0.04^c^	26.59 ± 0.02^d^	25.33 ± 0.06^e^	24.16 ± 0.05^f^
a*	14.79 ± 0.01^a^	14.76 ± 0.04^a^	11.78 ± 0.05^b^	11.15 ± 0.03^c^	8.35 ± 0.12^d^	8.13 ± 0.05^e^
b*	40.82 ± 0.07^a^	26.71 ± 0.02^b^	18.75 ± 0.15^c^	14.89 ± 0.09^d^	9.24 ± 0.23^e^	8.81 ± 0.2^f^
Firmness (N)	7.41 ± 0.23^a^	7.08 ± 0.11^b^	5.13 ± 0.1^c^	3.56 ± 0.18^d^	2.85 ± 0.13^e^	2.1 ± 0.11^f^
Glucose (%)	1.58 ±0.21^f^	2.74 ± 0.10^e^	4.66 ± 0.04^d^	7.26 ± 0.27^c^	8.48 ± 0.09^b^	10.18 ± 0.16^a^
Fructose (%)	1.18 ± 0.13^f^	3.30 ± 0.41^e^	5.47 ± 0.31^d^	9.01 ± 0.73^c^	10.73 ± 0.16^b^	13.05 ± 0.28^a^
Sucrose (%)	76.91 ± 1.49^a^	55.05 ± 0.06^b^	35.84 ± 0.49^c^	24.50 ± 1.08^d^	20.64 ± 0.18^e^	20.24 ± 0.17^e^

Note

The amounts given in this table show the average data ± standard deviation obtained from 3 repeats. Dissimilar letters in each row show a significant difference at the level of 0.05.

The water activity of the samples compared to the control sample has increased significantly (*p* < 0.05) probably due to the increase of jujube powder in the formulation as well as an increase in the moisture content of the toffee samples. Additionally, it can be due to the presence of fructose in jujube powder that absorbs water (Ahmadnia & Sahari, 2008). On the other hand, sugar reduces water activity (Habibi Najafi et al., 2011), which can be clearly observed in the control formula. As shown in Table 3, the control sample has the least water activity.

The statistical analysis of fat content showed that, apart from Sample I, which did not show any significant difference with the control sample (*p* > 0.05), other toffee samples were significantly different with each other and also with the control sample in terms of fat content (*p* < 0.05). The decrease observed in the fat content might have been caused due to the increase of jujube powder in the formulations. Considering the fact that the fat content of jujube is very low (0.45%), the fat content of all formulations is the same; since the amount of the jujube powder increased from formula I to formula VI, accordingly the fat content increased, as well.

Oxidation is a chain chemical reaction that happens in unsaturated compounds like oils. This causes the toffees to lose their chemical quality (Samsiah, Moey, Azizah, & Latifah, 2009). In addition, these days, everybody is trying to lose weight and people care about their health more than before. Hence, they tend to consume low‐fat foods so that they can prevent obesity and its probable consequences. Therefore, they prefer low‐fat and low‐energy foods (Rezende, Benassi, Vissotto, Augusto, & Grossmann, 2015). Thus, reduction of fat and increasing the proportion of bioactive compounds made with fruits such as jujube in producing toffee is a positive characteristic.

In the present study, raw energy decreased, and none of the samples showed a significant difference in case of energy; no significant difference observed among these samples (control, formula II, III, and IV). Samples of formula V and VI represented significantly the lowest value of energy. This decrease can be justified in a way that for each gram of fat, 9 kilocalories and for each gram of sugar, 4 kilocalories are produced. Since, in the formulations of toffees, with increasing the amount of jujube powder, the amount of sugar and fat is reduced, thus, the energy decreases (FarzanMehr, Abbasi, & Sahari, 2008).

According to the findings, as the amount of jujube powder increased, the total ash content in the samples increased, too, and the samples did not show any significant difference with each other at *p* < 0.05 level. Habibi Najafi et al. (2011) reported that by adding permeate, which is full of minerals, to toffees, the ash content in the samples containing permeate increased compared to the control sample. Accordingly, it is inferred that the reason behind the increase of the ash content in the toffee samples in this study is the presence of minerals in jujube. Small increase (1%) observed in the toffee samples’ ash content is due to the low total ash content of jujube powder (2%).

Table 3 shows a little increase in the level of proteins in the toffee samples as the amount of jujube powder increases. However, the difference was not meaningful at the level of 0.05. The only sample that showed a meaningful difference (*p* = 0.05) with the other samples was sample I (control) which had the lowest protein. The findings on the protein content in the toffee samples in this study are similar to those of Gehlot, Singh, and Siddiqui (2010). In 2010, Khapre studied the effects of adding different levels of guava pulp and soybean biomass to the formulation of the toffee samples. The protein content of the ultimate product was reported to be 6.4%, which showed an increase compared to the control sample. This had happened because the soybean biomass contained a lot of protein. In this study, the increase of protein in the toffee samples by nearly 3% corresponds to the protein content in the jujube powder (3.6%).

The pH test results in Table 3 showed that samples I to V do not differ meaningfully at the level of 0.05, but sample VI differs meaningfully with the others. The reason for the differences observed between the pH of the samples is the presence of pectic sugars (polygalacturonan and ramnugalacaturonan) in the jujube powder (Zhao et al., 2006). As the amount of jujube powder increases, the pH in each sample decreases meaningfully at the level of 0.05. Pectic polysaccharides are acidic, and they have carboxyl groups in their structures; hence, they decrease the pH of the samples.

Table 2 indicates that the overall amount of glucose and fructose (invert sugar; 17.82% + 21.85% = 39.67%) in the jujube powder is more than sucrose (33.89%). It is also seen in Table 3 that sucrose in the samples produced from the formulas I to VI decreases and the amount of replaced jujube powder increases, but fructose and glucose increase meaningfully at the level of 0.05 (*p* < 0.05).

The findings on the color indices of a*, b*, and L* in the toffee samples (Table 3) showed a meaningful difference at the level of 0.05. The indices L* (transparency), a* (redness), and b* (yellowness) are decreased as the level of jujube powder increases. This finding corresponds to the results reported by Ahmadnia and Sahari (2008). Reduced transparency of the produced toffee is due to the reaction of Maillard and Strecker among the proteins of milk powder with the reducing sugars of glucose and fructose in the jujube powder and lactose in the milk powder. Temperatures 100–150°C during the processing of toffee accelerate the non‐enzymatic browning reactions. A glance at the decreasing trend of a* and b* shows that the decreasing trend of b* is faster (b* has decreased from 41 to 9, and a* has decreased from 15 to 8). According to the findings given in Table 2, it is understood that a* and b* in the jujube powder decrease by 7 (15 − 8 = 7) and 12 (41 − 29 = 12) units, respectively, compared to the control sample (without any jujube powder). This proves that the toffee matrix and its compounds are effective in decreasing the indices of colors. The b*, a*, and L* indices have a direct relationship with light absorption, which in turn depends on compounds of the food matrix. The increase in the amount of jujube matrix in the formulations, which are mostly yellow or orange compounds, leads to the diffraction of scattering light and thereby causes a decrease in the a* and b*indices (Fennema, 2005).

As it is seen in Table 3, the more sugar is replaced with the jujube powder, and the toffee samples become less stiff significantly (*p* < 0.05). The findings of a previous research showed that the size of raw materials plays a crucial role in the firmness of the texture, and there is an inverse relationship between them (Afoakwa, Paterson, Fowler, & Vieira, 2009). It seems that the size of the jujube powder particles plays a more important role in the stiffness of the texture compared to other raw materials. As the particles have been sifted using a 70 mesh, these particles have been effective in forming porous tissues and reducing the firmness of the texture, because they are bigger in size compared to the other particles.

Comparison of the findings on the firmness and moisture of the samples shows that moisture of the samples has a negative correlation with their firmness, and as the moisture increases, the firmness is reduced, which is not unexpected. In some sources, moisture has been considered as the most effective element in determining the firmness of the samples (Stansell, 1995).

**Figure 2:**
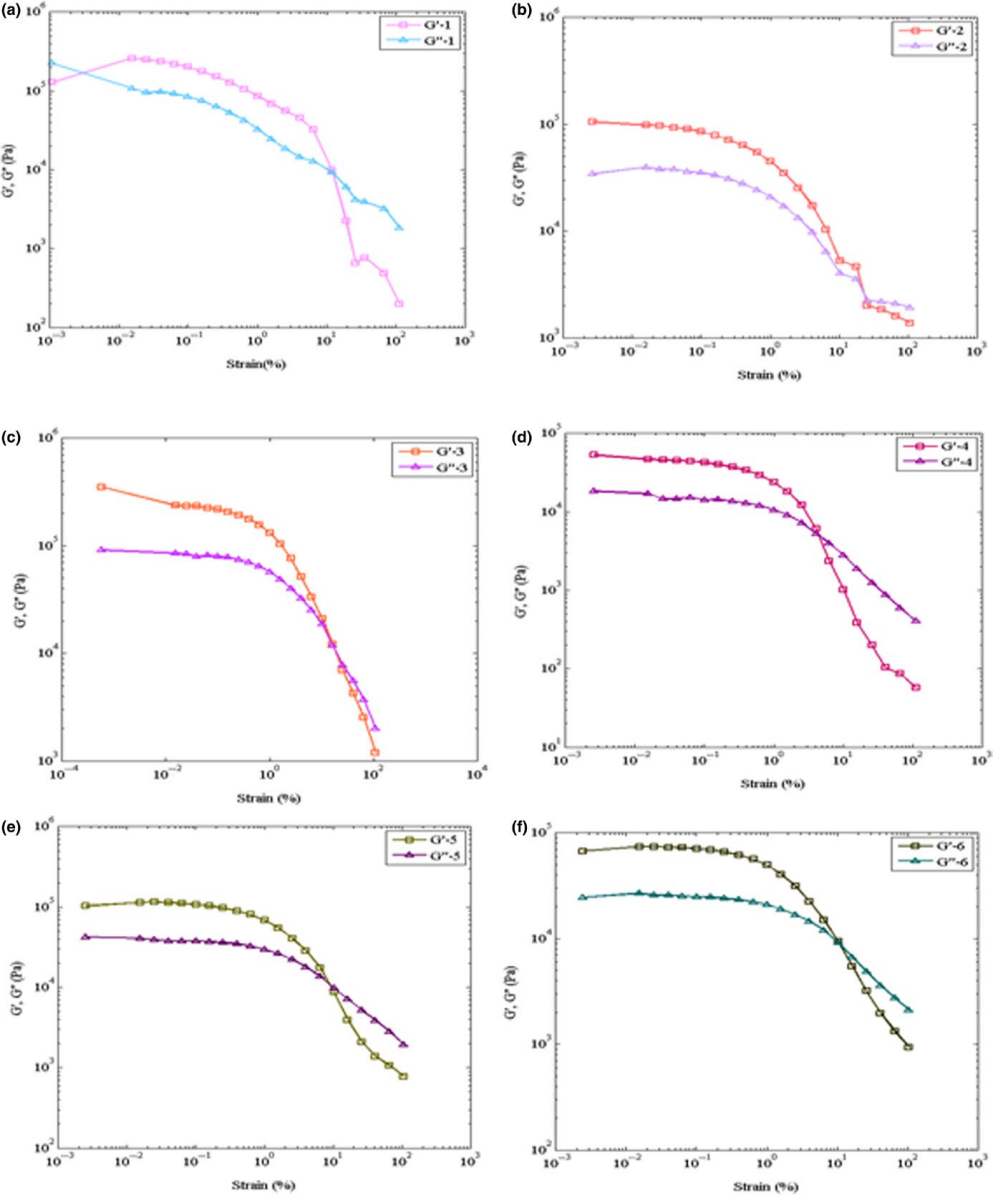
Stress sweep test storage modulus (*G′*) and loss modulus (*G″*); the control formula I figure a_1_ (*G′*‐1, *G″*‐1), Formula II figure b_1_ (*G′*‐2, *G″*‐2), formula III figure c_1_ (*G′*‐3, *G″*‐3), formula IV figure d_1_ (*G′*‐4, *G″*‐4), formula V figure e_1_ (*G′*‐5, *G″*‐5), and formula VI figure f_1_ (*G′*‐6, *G″*‐6) at 45°C and 0.5 Hz frequency

**Figure 3:**
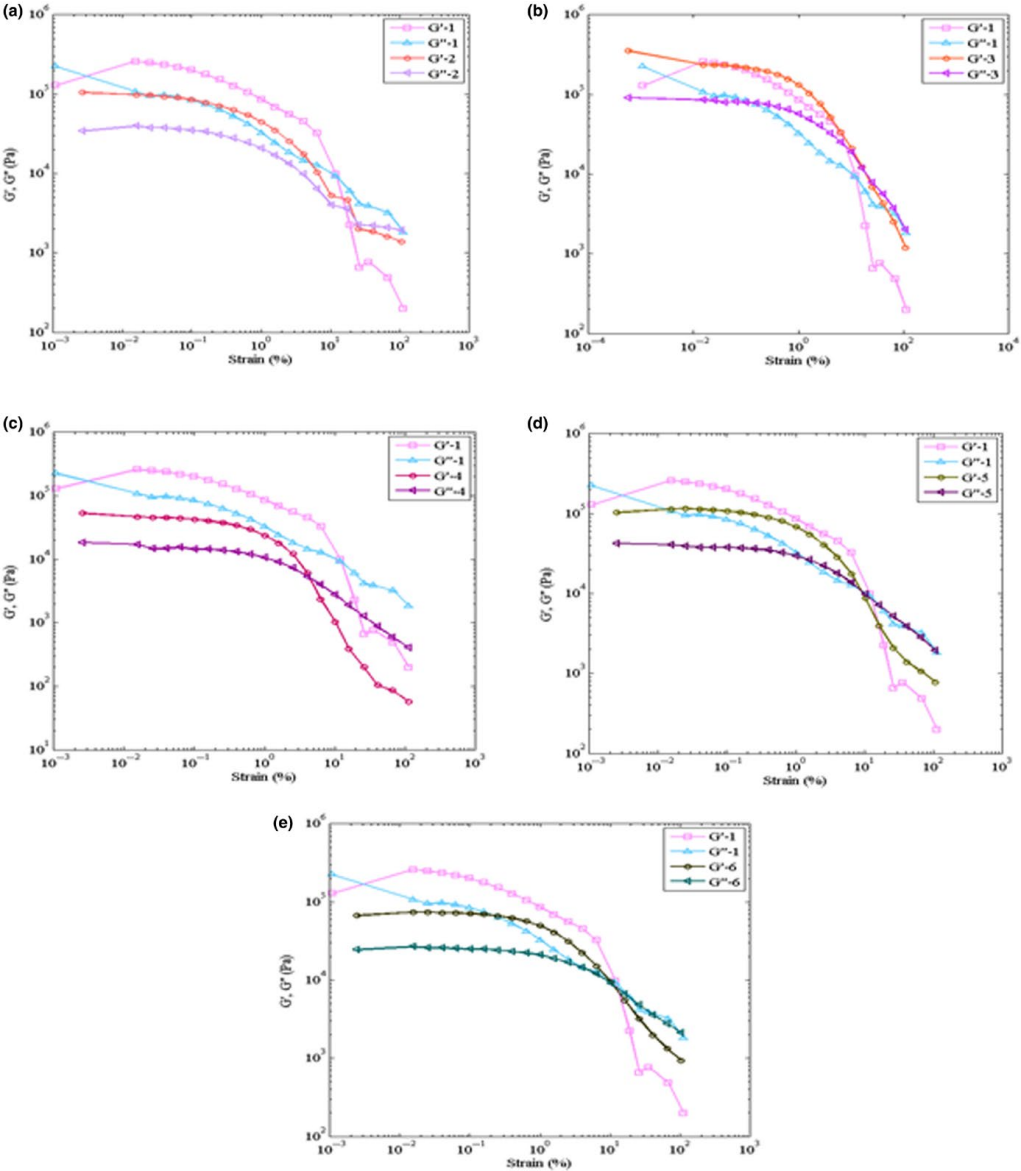
Comparison of the viscoelastic behavior of the toffee samples; formula II (*G′*‐2, *G″*‐2) with the control formula (*G′*‐1, *G″*‐1) figure a_2_, formula III Figure (*G′*‐3, *G″*‐3) with the control formula (*G′*‐1, *G″*‐1) figure b_2_, formula IV (*G′*‐4, *G″*‐4) with control formula (*G′*‐1, *G″*‐1) figure c_2_, formula V (*G′*‐5, *G″*‐5) with the control formula (*G′*‐1, *G″*‐1) figure d_2_, and formula VI (*G′*‐6, *G″*‐6) with the control formula (*G′*‐1, *G″*‐1) figure e_2_

The increase in jujube powder with 7% primary moisture content (Table 2) caused the increase in moisture content of the samples (Figures 2 and 3).

The results of rheological test on the toffee samples are shown in Figures 1 and 2. Oscillatory tests were performed in dynamic conditions to evaluate *G′* and *G″* and investigate the viscoelastic properties of samples. Stress sweep test was done at the fixed frequency of 0.5 with the diameter of 1 mm. In the stress sweeping test, the linear viscoelastic region is a region in which *G′* and *G″* are fixed when *G′* and *G″* are equal (*G′ *= *G″*); in other words, when they intersect, the materials start to flow; this point is called the flow point. After the flow point, *G′* and *G″* start to decrease with the increase of the strain; this region is a non‐linear region. The stress sweeping test is done at a fixed frequency and changeable strain. If *G′* > *G″*, the material will show a specific firmness in the test, which is mainly used for solid and sturdy dough. On the other hand, if *G′* <* G″*, it means that the behavior of the liquid is similar to the behavior of the material (Balaghi, Mohammadifar, Zargaraan, Gavlighi, & Mohammadi, 2011).

Oscillatory testing is the most prevalent method for studying the viscoelastic behavior of the foods. The results of this method can be used for studying the chemical compounds and the physical structure of the materials. In order to determine the viscoelastic behavior characteristics of the samples, Storage (*G′*) and Loss (*G″)* modulus are used (Angioloni & Collar, 2009; Samavati, Emam‐Djomeh, Mohammadifar, Omid, & Mehdinia, 2011). The storage modulus shows the magnitude of the stored energy in the material, and the loss modulus shows the magnitude of the energy wasted. Hence, for a full elastic material, which stores the total applied energy, *G″* is zero. In a liquid, which does not have any elastic characteristics, all the energy is wasted as heat, and *G′* will be zero. Foods are something between the two modes of elastic and viscose, called viscoelastic (Azarikia & Abbasi, 2010).

As shown in Figure 1, in the toffee samples I to VI, *G′* > *G″* implies the elastic behavior of the produced toffee samples. The comparison between the elastic behaviors of the toffee samples II to VI with the control sample is shown in Figure 2; it is observed that *G′* and *G″* values in the control sample are higher than those in the other samples. This shows that, as the jujube powder level increases, the elastic behavior of the sample decreases. As mentioned earlier in the texture test, the firmness decreases as the amount of jujube powder increases. The results obtained from the rheological test correspond to those obtained from the firmness test of the texture.

As it is seen in Figure 2, the elastic behavior of samples III and V was closer to that of the control sample; that is, the amounts of *G′* and *G″* in samples III and V were closer to that of the control sample. Thus, toffee samples III and IV were chosen as the optimized formula for evaluating the sensory characteristics of the toffee samples.

### 3.2 Sensory evaluation results

Among the six formulas of toffees, the control sample and the samples containing 40% and 60% jujube powder were chosen for sensory analysis. The reason why the sample containing 20% jujube powder was not chosen was that it cannot be used as a functional diet product. The levels of 80% and 100% were not chosen because they have after‐taste of jujube. As mentioned before in the rheological test section, the two levels of 40% and 60% were chosen as the optimized levels and then were tested in terms of the sensory characteristics. The toffee samples with code A (control sample), B (containing jujube powder replaced with 40% of sugar), and C (containing jujube powder replaced with 60% of sugar) were identified, and then along with a questionnaire were given to 30 panelists (male and female), who were students of the Agricultural College and had passed the required trainings. The trainings were done according to the characteristics in the evaluation forms. The definitions and the important points for the measurable characteristics were also presented. The participants were asked to rate the qualitative characteristics such as the texture, chewiness, color, smell, taste, sweetness, and general acceptance of the product from I to V (V for the best quality and I for the least quality).

First of all, the normality of the data was tested using a parametric test in which the data were abnormal statistically. Then the sensory test of the samples was done using Kruskal–Wallis non‐parametric test. The findings showed that the toffee samples had a very little difference in terms of texture, chewiness, color, smell, taste, sweetness, and total acceptance of the product, but it was not statistically meaningful (*p* > 0.05). Thus, the statistical results were not presented. This shows that the sensory characteristics of the toffees have been accepted by the consumers. Sensory analysis of toffee samples (mean score) evaluated with 30 panelists is shown in Table 4.

**Table 4:** Mean score of sensory evaluation in studied toffee samples

Parameters	Formulation		
	Formula I (Control: jujube powder instead of 0% sugar)	Formula III (jujube powder instead of 40% sugar)	Formula IV (jujube powder instead of 60% sugar)
Texture	43.47^a^	45.70^a^	47.33^a^
Chewiness	46.92^b^	40.53^b^	48.05^b^
Taste	43.33^c^	42.00^c^	45.17^c^
Odor	43.30^d^	43.80^d^	45.40^d^
Sweet	42.90^e^	43.22^e^	47.38^e^
Color	47.65^f^	43.27^f^	45.58^f^
After‐taste	42.33^g^	42.68^g^	46.48^g^
General acceptance	42.33^h^	43.78^h^	47.38^h^

Note

The amounts given in this table show the average data obtained from 30 panelists. Similar letters in each row show not significant difference at the level of 0.05.

## 4 CONCLUSION

In this study, toffee produced by jujube powder instead of different levels of sugar and then physico‐chemical, rheological, and sensory characteristics were investigated. The results of toffee samples showed that the fat, raw energy, pH, color (L*, a*, b*), and hardness of samples were decreased by increasing percent of jujube powder in formulas but the moisture, water activity, ash, and protein content of toffee samples were increased with increasing percent of jujube powder. The results of sugar test showed that the content of glucose and fructose was increased and the sucrose content was decreased by increasing of jujube powders. The results of rheological test showed that storage modulus (*G′*) was greater than loss modulus (*G″*), which represents the elastic behavior (solid‐like) of samples, and storage modulus (*G′*) of control sample was greater than all samples; on the other hand, the hardness of tissue was decreased by increasing the percentage of jujube powder. According to physico‐chemical and rheological results, the optimum samples (III and IV = jujube powder instead of 40% and 60% sugar, respectively) were selected among 6 formulas of toffee. Then the sensory properties of their samples were compared with control. The results did not show a significant difference because all of three formulas were accepted by panelists.

## CONFLICT OF INTEREST

The authors notify that there are no conflicts of interest.

## ETHICAL STATEMENTS

This study does not involve any human or animal testing.
